# Psychological distress, anxiety, depression, stress level, and coping style in HIV-pregnant women in Mexico

**DOI:** 10.1186/s40359-023-01416-8

**Published:** 2023-11-01

**Authors:** María del Pilar Meza-Rodríguez, Blanca Farfan-Labonne, Miroslava Avila-García, Ricardo Figueroa-Damian, Noemí Plazola-Camacho, Gabriela Pellón-Díaz, Braulio Alfonso Ríos-Flores, Efraín Olivas-Peña, Phillipe Leff-Gelman, Ignacio Camacho-Arroyo

**Affiliations:** 1 Coordinación de Investigación en Psicología, Instituto Nacional de Perinatología, Montes Urales 800, CDMX, C.P. 11000 México; 2 Departamento de Fisiología y Desarrollo Celular, Instituto Nacional de Perinatología, Montes Urales 800, CDMX, C.P. 11000 México; 3 Departamento de Infectología e Inmunología, Instituto Nacional de Perinatología, Montes Urales 800, CDMX, C.P. 11000 México; 4 Departamento de Neurociencias, Instituto Nacional de Perinatología, Montes Urales 800, CDMX, C.P. 11000 México; 5 Departamento de Investigación en Salud Reproductiva y Perinatal, Instituto Nacional de Perinatología, Montes Urales 800, CDMX, C.P. 11000 México; 6 Unidad de Investigación en Reproducción Humana, Instituto Nacional de Perinatología-Facultad de Química, Universidad Nacional Autónoma de México, Montes Urales 800, Lomas de Chapultepec Miguel Hidalgo, Ciudad de México, C.P. 11000 México

**Keywords:** High-risk pregnancy, HIV, Depression, Anxiety, Coping style.

## Abstract

**Purpose:**

To evaluate the presence of psychological distress (PD) and its association with the mental health and coping styles of pregnant women living with HIV (PWLWH).

**Method:**

An observational, cross-sectional descriptive study was performed. Seventy-three PWLWH were included. Patients responded to a psychometric battery for PD, depression, anxiety, stress, and coping style evaluation. The scales used in the study were: Goldberg’s 30-item General Health Questionnaire (GHQ-30), State-Trait Anxiety Inventory (STAI), Zung Depression Self-Measurement Scale (ZDS), Nowack Stress Profile, Lazarus and Folkman’s Coping Styles Questionnaire.

**Results:**

PD was observed in 31.5% of the participants. PD-positive patients showed a higher probability of presenting traits of depression and anxiety and medium/high stress levels. Besides, they preferentially used emotion-focused coping styles.

**Conclusion:**

PD is associated with a higher probability of presenting anxiety and depression in PWLWH. Emotion-focused coping style could be a factor in decision-making associated with risk behaviors in PWLWH.

## Background

Human immunodeficiency virus (HIV) infection is a chronic degenerative disease [1]. Since the beginning of the epidemic, it has affected 79 million people [2], and its incidence during 2021 was 1.5 to 2 million people, with a prevalence of 37.7 million people [2]. Mexico presents 10,000 new annual cases. The gender gap indicates that 2/10 new cases correspond to women, and most (3 out of 4), are of reproductive age. Given that the main transmission route is sexual intercourse [3], pregnancy becomes a risk factor to be considered in this population [4]. Besides physical comorbidities, HIV seropositive individuals present psychological alterations. They show a higher depression and anxiety incidence than seronegative people [2].

Pregnancy and motherhood constitute a multifactorial maternal challenge, and HIV infection adds an assumed risk for children’s and women´s health. Physical and psychosocial development is modified [5–7] because it supposes increased responsibilities and difficulties that induce the appearance of emotional alterations associated with managing a socially stigmatized chronic disease.

HIV infection shades pregnancy and confuses women’s thinking among a range of emotional manifestations that confront them with the impossibility of achieving several social ideals [8]. These resignations lead women to experience a variety of fluctuating emotional states that might put at risk the mother´s effective role during pregnancy and rearing [9–12]. The emotional experience could influence health in multiple pathways, ranging from symptom recognition by choosing healthy or unhealthy behaviors as an emotional regulation strategy [13].

It is necessary to contemplate psychiatric alterations, mainly depression and anxiety, associated with perinatal complications such as preeclampsia, intrauterine growth retardation, and altered immune status [13–20]. Clinical evidence has shown that a better psychologically adapted patient improves tolerance to treatment and presents higher adherence rates, which undoubtedly influences pregnancy development [21].

Pregnant women’s initial attempts to cope with their condition could restrict their psychological resources, promoting a set of temporary emotional disturbances known as psychological distress (PD) [19, 22, 23]. PD refers to emotional changes related to a specific event; it is considered a reactive process without effects on personality structure. Patients experience subjective discomfort, decreased ego functions, and temporary restriction of consciousness [24]. This adaptive event includes psychic pain that triggers changes in the perception of affective tonality, mood, and thought, which alter daily performance.

Concern about pregnancy development might depend on each woman´s coping style (CS) [25], which is considered as the behavior and cognitive response related to a stressful circumstance. The person facing a stressful event performs two types of valuations; the first is aimed at checking if the event is threatening, and the second is focused on how to deal with it [26]. Stress arises when the solution to the problem exceeds emotional resources, demanding a cognitive, emotional, and physiological response [27, 28].

We aimed to evaluate PD’s association with depression, anxiety, and stress levels in pregnant women living with HIV (PWLWH) in Mexico. We also studied CS use in these patients.

## Materials and methods

A cross-sectional, observational, multivariate study was carried out in third-trimester PWLWH without mental retardation history. All women signed an informed consent to participate in the study. Their participation consisted of answering a semi-structured clinical history and a psychometric battery applied by a clinical psychologist. INPer in México authorized this study through its Ethics and Research Committees (No. 2017-3-128). The sample size was calculated by the proportion estimation formula (Zα = 1.96, p = 5%, d = 0.05) for 70 patients.

PD presence was assessed employing the Goldberg General Health Questionnaire in its 30-item version, validated in HIV-positive women [29]. This instrument explores the inability to function at a normal level perception from the psychic point of view and the psychic distress appearance [30]. A score above seven indicates PD presence [31].

The trait-state anxiety inventory (STAI) developed by Spielberg was used to evaluate the presence of trait anxiety as part of the personality structure and the transitory emotional situation. The cut-off point for pregnant women is 47 for trait anxiety and 43 for state anxiety [32].

The Zung Depression Self-Measurement Scale was chosen for the symptomatic quantification of depressive traits; it consists of twenty statements that relate to depression [33]. The minimum score is 20, and the maximum is 80, establishing 44 as the cut-off point for depressive traits in the obstetric population [34].

The Stress level was assessed utilizing the Nowack stress profile, designed to identify factors that allow tolerating the harmful consequences of everyday stress and those that make someone vulnerable to stress-related illnesses [35]. According to the standardization for the Mexican population, scores above 60 are considered severe; between 41 and 59, moderate; and below 40, mild [28].

Coping was measured employing the Lazarus and Folkman, coping styles instrument consisting of 67 items measuring eight coping styles: confrontational, distancing, self-control, social support, responsibility, escape-avoidance, problem-solving, and positive reappraisal [36, 37].

Additionally, once the women had their babies, we checked their medical records to know their contraceptive selection.

Data analysis. Statistical analysis was executed using the SPSS version 24 statistical package. For sociodemographic variables, descriptive statistics were achieved. The medical variables were analyzed employing the Chi-square test. Furthermore, Odds Ratios (OR) were determined.

## Results

Seventy-three pregnant women with HIV underwent prenatal care at the INPer HIV clinic in México. The mean patient´s age was 27.7 years. Their marital status was predominantly civil union with their partner or single cohabiting with their immediate family; the mean time since HIV diagnosis was 5.3 ± 4.7 years (Table 1).

**Table 1:** HIV-Pregnant Women Demographic Characteristics

Demographic characteristics	Frequency %
**Maternal age (years)**	
Less 16	2.7
17–22	24.6
23–28	27.4
29–34	28.7
35–40	12.3
41–46	4.1
**Scholarship**	
Elementary school	28.7
Junior high school	36.9
Senior high school	24.6
College	9.5
**Marital status**	
Single	36.6
Married	21.9
Free union	36.9
Widow	5.4
Divorced	0.0
**Economical activity**	
Home	71.2
Commercial	10.9
Employee (private)	10.9
Employee (domestic)	1.3
Under employee	1.3
Student	2.7
Unemployed	1.3
**Religion**	
Chatolic	83.3
Christian	12.3
Atea	1.3
**Cohabitation**	
Direct family (children, siblings, and partner)	58.9
Extended direct family (partner, children, parents, siblings, and grandparents)	27.4
Indirect family (uncles, aunts, in-laws)	13.7
**Family dinamic**	
Adequate	75.3
Regular	19.1
Bad	5.4
History of sexual abuse	10.9

Regarding their gynecological history, this group of patients had 2.96 ± 2.4 sexual partners (Table 2). The average number of pregnancies was 2.3±1.2, and 21 patients were in their first pregnancy (Table 2). In all cases, pregnancy was unplanned.

**Table 2:** Gynecologist and obstetric history of HIV pregnant women

Gyneco-obstetric characteristics	Mean±*SD* proportion n = 73
**Maternal age (years)**	27.70±7.0
Less than 16	2/73
17–22	18/73
23–28	20/73
29–34	21/73
35–40	9/73
41–46	3/73
**Beginning of sexual life**	17.60±3.0
Less than 15	17/73
16–24	53/73
25–28	3/73
**Number of sexual partners**	2.96±2.4
1–3	55/73
4–7	14/73
8–11	3/73
12–15	1/73
**Obstetric History**	
Gestation	2.30±1.2
Births	1.10±1.2
Caesarean section	0.45±0.8
Abortions	0.12±0.4
Stillbirths	0.04±0.2
**Parity**	
Nulliparous	21/73
One child	27/73
Several children	25/73

Concerning family planning methods, before pregnancy, 44/73 women reported the use of a contraceptive method, with male condoms being the most common (39/73). By the end of their pregnancy, 49/73 opted for a definitive method, predominantly bilateral tubal occlusion (BTO) (Table 3).

**Table 3:** Choice of contraceptive methods prior to pregnancy and after childbirth

Contraceptive method	Pregestational n = 73	Postpartum n = 73
**Family planning method**	44/73	63/73
Condom	39/73	5/73
Intrauterine device	2/73	7/73
Oral contraceptive	1/73	0/73
Muscle contraceptive	2/73	0/73
Subdermal hormonal implant	0/73	2/73
Bilateral Tubal Obstruction(OTB)	0/73	49/73
None	29/73	10/73
**Temporal/Definitive Family planning method**		
Temporal	44/73	14/73
Definitive	0/73	49/73
None	29/73	10/73

The predominant HIV transmission route was the sexual intercourse (64/73). Twenty-six patients are still in a relationship with the partner who infected them, while 40/73 are related to a different partner. In both cases, they keep a relationship with the parents of the children they are expecting (Table 4).

**Table 4:** Infectious history concerning HIV.

HIV history	Mean±SD Proportion n = 73
**Mother’s route of transmission**	
Sexual	64/73
Vertical	7/73
Transfusion	2/73
**Time of diagnosis (years)**	5.29±4.8
Less than 1	15
1–3	20
4–6	9
7–9	14
10–12	9
13–15	3
16–18	3
**Viral load at diagnosis (copies/mL)**	1.38±0.5
Undetectable (< 50,000 copies/mL)	45
Detectable (> 50,000 copies/mL)	28
**Viral load at pregnancy (copies/mL)**	1.25±0.4
Undetectable (< 50,000 copies/mL)	55
Detectable (> 50,000 copies/mL)	18
**Seropositivity in previous offspring**	9/73
**Reported knowledge of current partner´s seropositivity**	71/73
**Cohabitating with the infecting partner**	26/73

### Psychological distress

When comparing mental health parameters from women with (n = 29) and without (n = 44) PD, results indicated that 25/29 women with PD had depressive traits (Chi-square, *p < 0.001*), and 21/29 exhibited medium-high stress levels (Table 5). Pregnant women with PD presented higher trait-anxiety (17/29) (Chi-square, *p < 0.001*) and state-anxiety (24/29) (Chi-square, *p < 0.001*) than those without PD.

**Table 5:** Psychological characteristics of pregnant women with and without psychological distress

Characteristic		With PD n = 29	Without PD n = 44	*P value*
Depression	Positive	25	18	0.0001*
	Negative	4	26	
Stress level	Low	8	28	0.004*
	Medium	16	15	
	High	5	1	
Anxiety-state	Positive	24	6	0.0001*
	Negative	5	38	
Anxiety-trait	Positive	17	6	0.0001*
	Negative	12	38	

*Chi-square, *P value* < 0.05

An OR analysis was performed between the psychological variables, finding that cases with PD have 9.03 times the probability of presenting depression (IR 95%; 2.68–30.41), 8.97 for trait-anxiety (IR 95%; 8.35-110.68), 4.59 for medium-high stress levels (IR 95%; 1.66–12.74), and 4.47 fair to low-income family dynamics (IR 95%; 1.438–13.90) than patients without PD.

Patients with PD were more likely to choose a contraceptive method once the pregnancy had resolved than patients without PD, who maintained contraceptive use behavior (OR 2.5, IR 95%, 0.348–17.94). Fifty-three percent (39/73) of the patients who previously used contraceptives changed from a temporary to a permanent method.

Regarding associations between alcohol, drug use, and sociodemographic characteristics, most patients denied alcohol or drug consumption. However, alcohol or drug consumption in their partners was 14/73 and 11/73, respectively. Pregnant women without PD with partners without alcohol or drug consumption history showed a higher proportion of a good family relationship perception (26/30, Chi-square, *P = 0.005*).

### Coping style

Some emotion-focused coping styles were higher in PD HIV women than in the control group: self-control (*p < 0.001*), responsibility (*p = 0.018*), and escape avoidance (p *< 0.001*) (Table 6). Likewise, confrontational coping, a problem-based style, was higher in PD HIV women.

**Table 6:** Coping style of pregnant women with and without psychological distress

	Mean ± *SD*	*P value*
**Self-control**		
With PD	1.49 ± 0.4	0.001*
Without PD	1.07 ± 0.6	
**Responsibility**		
With PD	1.76 ± 0.8	0.018*
Without PD	1.28 ± 0.5	
**Escape-avoidance**		
With PD	1.33 ± 0.5	0.001*
Without PD	0.74 ± 0.5	
**Confrontational**		
With PD	1.50 ± 0.6	0.004*
Without PD	1.23 ± 0.5	

* T-test, *P value* < 0.05

## Discussion

It has been described that HIV infection profoundly affects women’s mental health [38]. When pregnancy occurs in a woman living with HIV, the psychosocial and clinical conditions associated with mental health deterioration can be further increased. Despite this, few studies seek to delve into this issue [39, 40].

Koniak-Griffin (1994) describes that even knowing their HIV infection, most pregnant women in the USA minorities (Latinas and African-Americans) exhibited unprotected sex and other risk-taking behaviors [41]. Gómez Suarez (2016) showed that approximately 38% of unplanned pregnancies occur in the general population because of unmet contraceptive needs, but this number tripled in women living with HIV [42]. These risk-behavior patterns are similar to those in our population; 39.7% of the women studied did not use any contraception method and 53.4% only used a temporary method such as a condom.

Scientific evidence points out a perception of a submissive position and the conception of an inferior role in affective-sexual relationships in women; this inequality has been observed in non-pregnant and PWLWH [43, 44]. Bertagnoli and Figueiredo (2017) identified a difficulty in power distribution as a structural element of vulnerability in HIV-women relationships [45]. These misconceptions may lead to neglected self-healthcare associated with a shorter survival time than men with AIDS [46, 47].

The del-Romero and collaborator´s study (2004) points out that in HIV-positive men, 40% did not use condoms regularly; furthermore, 19% reported having accidents using condoms [48]. Consequently, many of their couples became pregnant [42–44, 46–48]. In our population of PWLWH, the patterns of behavior described in the del-Romero study were repeated, given that 87.6% of our patients were sexually infected.

Alcohol and drug abuse are other risk behaviors in the HIV population (Rosemary’s study) [49]. Although the women in the study denied a history of alcohol or drug abuse, our results indicate that alcohol or drug abuse in the patient’s partners occurs in a proportion of 1 in 5 (fathers of the children they will give birth to). Considering that with adequate medication and clinical follow-up, the rate of vertical transmission is less than 2%, the high rates of positive serostatus of previous offspring (12%, 9/73) in these patients could be explained by the psychological characteristics of the mothers [50–52] and the risk-behavior pattern they display as a consequence.

Regarding the mental health of PWLWH, it has been suggested that a codependency pattern is associated with a higher probability of presenting secondary emotional disturbances [53]. Poorer family dynamics favor maladaptive behaviors that affect women emotionally [43, 44]. Our data showed higher proportions of depression among PWLWH (58.9%) than depression rates in women living with HIV reported in other studies (25–40%). Regarding anxiety, our outcomes of 31.5% were similar to anxiety rates reported for HIV-positive women (23–40%). Finally, our population’s stress levels are comparable to previously reported data (34.8%) [7, 38, 54–56].

Although several studies suggest that marital status plays a role in mental health [52, 55, 56], our results did not show differences in depression, anxiety, or stress levels between women who live with their partners and single women. In our sample, educational level neither influenced the proportion of depression nor anxiety; our results match the report of Ogueji and collaborators (2021) on the Nigerian population. The demographic characteristics from different social contexts may influence the development of mental health deteriorating in the PWLWH.

Additionally, inappropriate family and social relationships affect the development of pregnancy and the health of the maternal-fetal dyad. Previous research indicates that infants with absent fathers have an increased risk of unfavorable fetal birth outcomes, particularly in HIV-positive women [57].

The results of this study indicate that pregnant women related to partners with alcohol and drug abuse patterns presented a higher incidence of PD than those with abstemious partners. Substance abuse could affect family welfare since pregnant women without PD, whose couples had no alcohol or drug consumption history, showed better family relationships.

Previous studies have shown that familiar unfavorable contexts could correlate negatively with the development of pregnancy in PWLWH. Hatcher and coworkers reported that one-third of women with HIV in South Africa experienced intimate partner violence, getting a high pregnancy complications rate and a higher viral load [58]. These findings highlight the importance of partner relationships and the role of paternal involvement as a significant component of maternal and fetal health during pregnancy.

The fine-grained description of the factors influencing mental health is relevant since the PWLWH condition has high adverse psychological burdens. Women in this situation show higher risk behaviors on self-care, such as low treatment adherence or an increased proportion of suicidal thoughts, the last one that occurs in nearly 3% of the PWLWH. [59, 60]. These results emphasize the need for closer monitoring of the mental health of women with HIV and more well-defined psychological and psychiatric standards, particularly during pregnancy.

Gómez et al. (2016), studying a population of high-risk pregnant women different to HIV etiology, like gestational diabetes, hypertensive disease of pregnancy, or advanced maternal age, found that the main emotional variations were sadness, emotional devastation, and fear of pregnancy complications [19]. These conditions could lead to the appearance of PD.

The PD usually acts as an adaptive mechanism that could play a role of protective factor [58, 61] because it mobilizes personal resources in search of specialized help, depending on the psychological characteristics of women. In our study, one out of 3 pregnant patients presented PD.

This proportion was lower than PD reported in HIV-positive teenagers (48.2%) or in other studies of PWLWH, which report PD rate until 69% [54, 62].

Our observations suggest that PD is associated with medium-high levels of stress and increased levels of state anxiety and depression. These psychological conditions, added to inadequate family and social background, are associated with adverse pregnancy events [16, 18, 61, 63, 64]. HIV pregnancy entails a substantial change in the psychosocial spheres; therefore, these women present a higher risk for adverse obstetric events than those without HIV [65–67]. In addition, high levels of anxiety and depression increase the possibility of postpartum depression, which undoubtedly harms the newborn and affects the mother´s quality of life, interfering in the establishment of the binomial bond and newborn development [68–71].

Diverse studies suggest that maternal mental health care should extend beyond childbirth and should be accompanied by strategies that permit the woman to adapt to the new challenges according to her psychological characteristics, including her coping style. According to the coping theory, styles that direct their efforts towards emotion are more likely to appear when the resolution perception is limited; therefore, emotions tend to predominate in the response pattern, moving away from the rational plane [26, 72]. According to this theory, PD could increase the scores for emotion-focused styles. Moreover, the scores for the confrontational style are higher in the PD group than in women without PD. The confrontational style is most effective when a stressful event persists over time or in a long-duration process like pregnancy.

It has been demonstrated that in the HIV-positive population, avoidance strategies are predictors of emotional distress that can function as a protector in help-seeking [73]; we propose that this need for help should be at least partly met by the medical services, given that social and family conditions are not always adequate.

Our findings on contraception method choice suggest that the hospital context and counseling influenced self-care behaviors of the PWLWH by increasing the rate of acceptance of a contraceptive method. Medical care could contribute to more reasoned decision-making and trigger the mobilization of adaptive resources, specifically concerning the decision to avoid a new pregnancy. These data imply the change in the decision to use a permanent contraceptive method, as it was observed in our population.

Finally, independent of PD presence, our results indicate that coping style could influence contraceptive choice. When we analyzed the coping pattern as problem-based or emotion-based according to contraceptive choice after the resolution of pregnancy, it was observed that women with higher scores for problem-based coping style selected a permanent contraceptive. More research is needed to understand how coping styles could influence women’s decisions and how clinicians could prepare better strategies according to coping style to affront HIV-pregnancy health.

## Conclusions

According to the World Health Organization, HIV infection rates and AIDS cases among women have increased meaningfully in the last ten years. Given the risk behaviors of this population, it is expected that pregnancies in HIV-positive couples will occur. Our results indicate that PD is present in most PWLWH. These patients showed a higher probability of presenting traits of depression, anxiety, and elevated stress levels. In addition, our results indicate that PWLWHI preferentially used emotion-focused coping styles, which could be a factor in decision-making associated with their risky behaviors. It is necessary to establish new lines of clinical research focused on the factors influencing women’s mental health to establish services more responsive to women’s needs.

## Data Availability

The data and psychometric instruments are available.

## References

[1] Deeks SG, Lewin SR, Havlir DV (2013). The end of AIDS: HIV Infection as a chronic Disease. Lancet.

[2] UNAIDS. UNAIDS Data. 2021; Available from: https://www.unaids.org/es/resources/fact-sheet.

[3] WHO VIH in pregnancy, a review. 1998. 59.

[4] CENSIDA SdS. *Vigilancia Epidemiológica de casos de VIH/SIDA en México*. 2021 [cited 2022; Available from: https://www.gob.mx/cms/uploads/attachment/file/513720/RN_D_a_Mundial_sida_2019.pdf.

[5] Finocchario-Kessler S (2010). Understanding high fertility desires and intentions among a Sample of Urban Women Living with HIV in the United States. AIDS Behav.

[6] Conde Higuera P (2016). Estigma, discriminación y adherencia al tratamiento en niños con VIH y SIDA: Una Perspectiva bioética. Acta Bioethica.

[7] Ngocho JS (2019). Depression and anxiety among pregnant women living with HIV in Kilimanjaro region, Tanzania. PLoS ONE.

[8] Obiols MJ, Stolkiner AI. *Mujeres viviendo la maternidad con VIH/SIDA: la salud mental y el sosténcompartido de los cuidados* Perspectivas en Psicología: Revista de Psicología y Ciencias Afines, 2018. 15(2): p. 56–68.

[9] Legere LE (2017). Approaches to health-care provider education and professional development in perinatal depression: a systematic review. BMC Pregnancy Childbirth.

[10] Einloft Kleinibing R (2016). ESTRATÉGIAS DE CUIDADO À SAÚDE DE GESTANTES VIVENDO COM HIV: REVISÃO INTEGRATIVA. Ciencia Y enfermería.

[11] Piccinini C (2012). Perceptions and feelings of pregnant women concerning prenatal care. Psicologia: Teoria E Pesquisa.

[12] Carvalho FTd (2009). Intervenção psicoeducativa para gestantes vivendo com HIV/Aids: uma revisão da literatura. Psicologia: Teoria e prática.

[13] Meza-Rodríguez MP, FrMorales-Carmona M-RJ (2011). Adaptación psicológica en mujeres con infección por virus de papiloma humano. Perinatol Reprod Hum.

[14] Evans J (2001). Cohort study of depressed mood during pregnancy and after Childbirth. BMJ.

[15] Diego MA (2004). Prepartum, postpartum, and chronic depression effects on newborns. Psychiatry.

[16] Borders AEB (2007). Chronic stress and low Birth Weight neonates in a Low-Income Population of women. Obstet Gynecol.

[17] Lara MA, Navarro C, Navarrete L (2010). Outcome results of a psycho-educational intervention in pregnancy to prevent PPD: a randomized control trial. J Affect Disord.

[18] Accortt EE, Cheadle AC, Dunkel C, Schetter (2015). Prenatal depression and adverse birth outcomes: an updated systematic review. Matern Child Health J.

[19] Gomez-Lopez MA, Sugiyama BS (2016). Malestar psicológico en mujeres con embarazo de alto riesgo. Summa Psicol UST.

[20] Chinchilla-Ochoa D (2019). Depressive symptoms in pregnant women with high trait and state anxiety during pregnancy and postpartum. Int J Womens Health.

[21] Belmar J, Stuardo V (2017). Adherencia Al Tratamiento anti-retroviral para El VIH/SIDA en mujeres: una mirada socio-cultural. Revista Chil De infectología.

[22] Fisher JR, Feekery CJ, Rowe-Murray HJ (2002). Nature, severity and correlates of psychological distress in women admitted to a private mother-baby unit. J Paediatr Child Health.

[23] Glazier RH (2004). Stress, social support, and emotional distress in a community sample of pregnant women. J Psychosom Obstet Gynaecol.

[24] Espíndola Hernández JG (2006). Malestar psicológico: algunas de sus manifestaciones clínicas en la paciente gineco-obstétrica hospitalizada. Perinatología Y reproducción Humana.

[25] Furber CM (2009). A qualitative study of mild to moderate psychological distress during pregnancy. Int J Nurs Stud.

[26] Lazarus RS (1986). Estrés Y procesos Cognitivos.

[27] Vargas Castañeda N, García Casós CMJ, Marquez Leyva V. F *Stress, coping strategies and academic performance in nursing students of the National University of Trujillo* Revista Peruana: Enfermeria, investigación y desarrollo, 2013. 11(1): p. 57–66.

[28] De la Roca-Chiapas JM (2019). Validación Del Perfil de Estrés de nowack en estudiantes universitarios mexicanos. Revista De Salud Pública.

[29] Meza Rodríguez MdP (2014). Validación interna de un Cuestionario General De Salud (CGS – 30)en mujeres seropositivas al VIH. Revista Latinoam De Med Conductual / Latin Am J Behav Med.

[30] Goldberg DP, Hillier VF (1979). A scaled version of the General Health Questionnaire. Psychol Med.

[31] Morales-Carmona F, Barroso-Aguirre L-CM (2002). Alteraciones emocionales en una muestra de mujeres mexicanas con eventos ginecoobstétricos. Perinatol Reprod Hum.

[32] González G (1990). Normalización De Un instrumento de ansiedad (IDARE) en mujeres embarazadas. Revista Mexicana De Psicología.

[33] Zung WW (1972). The Depression Status Inventory: an adjunct to the Self-Rating Depression Scale. J Clin Psychol.

[34] González G (1993). Normalización De Un instrumento para medir depresión en mujeres embarazadas. Perinatol Reprod Hum.

[35] Meza Rodríguez MP (2018). Niveles De estrés en pacientes mexicanas embarazadas seropositivas al VIH. Perinatología Y Reproducción Humana.

[36] Folkman S, Lazarus RS (1988). Coping as a mediator of emotion. J Pers Soc Psychol.

[37] Zavala Yoe L, et al. Validación Del instrumento de estilos de enfrentamiento de Lazarus Y Folkman en adultos de la Ciudad De México. Volume 10. Revista Intercontinental de Psicología y Educación; 2008. pp. 159–82. 2.

[38] Levine AB, Aaron EZ, Criniti SM (2008). Screening for depression in pregnant women with HIV Infection. J Reprod Med.

[39] Faler CS (2016). Diagnóstico VIH-SIDA: Los Impactos causados en la persona en las relaciones y estructura familiar. Sal Jal.

[40] Vera PVE (2004). Influencia social y familiar en El comportamiento del pacientes con VIH/SIDA ante su diagnóstico y su manejo. Rev Hosp Jua Mex.

[41] Koniak-Griffin D (1994). Risk-taking behaviors and AIDS knowledge: experiences and beliefs of minority adolescent mothers. Health Educ Res.

[42] Gomez-Suarez M (2016). [Meeting contraceptive needs of HIV-positive women: effect on elimination of vertical transmission of HIV]. Rev Panam Salud Publica.

[43] Lopez A (2023). HIV Stigma mechanisms Scale: factor structure, reliability, and validity in Mexican adults. AIDS Behav.

[44] Wowolo G (2022). The impact of different parental figures of adolescents living with HIV: an evaluation of Family structures, perceived HIV related stigma, and opportunities for Social Support. Front Public Health.

[45] Bertagnoli M, Figueiredo M (2017). Gestantes Soropositivas Ao HIV: Maternidade, Relações Conjugais E Ações Da Psicologia.

[46] Lea A (1994). Women with HIV and their burden of caring. Health Care Women Int.

[47] Tiouiri H (1999). [Study of psychosocial factors in HIV infected patients in Tunisia]. East Mediterr Health J.

[48] del Romero J (2004). [Women who are partners of a man infected by HIV: description of their characteristics and appraisal of risk]. Aten Primaria.

[49] Wong M (2017). Depression, alcohol use, and stigma in younger versus older HIV-infected pregnant women initiating antiretroviral therapy in Cape Town, South Africa. Arch Womens Ment Health.

[50] Kafack EVF (2022). Evaluation of plasma viral-load monitoring and the prevention of mother-to-child transmission of HIV-1 in three health facilities of the Littoral region of Cameroon. PLoS ONE.

[51] Myer L (2017). HIV viraemia and mother-to-child transmission risk after antiretroviral therapy initiation in pregnancy in Cape Town, South Africa. HIV Med.

[52] Zijenah LS (2022). Impact of option B(+) combination antiretroviral therapy on Mother-to-child transmission of HIV-1, maternal and infant virologic responses to combination antiretroviral therapy, and maternal and infant mortality rates: a 24-Month prospective Follow-Up study at a Primary Health Care Clinic, in Harare, Zimbabwe. AIDS Patient Care STDS.

[53] Moseholm E (2022). Psychosocial health in pregnancy and postpartum among women living with - and without HIV and non-pregnant women living with HIV living in the nordic countries - results from a longitudinal survey study. BMC Pregnancy Childbirth.

[54] Qin S (2019). Survey and analysis for impact factors of psychological distress in HIV-infected pregnant women who continue pregnancy. J Matern Fetal Neonatal Med.

[55] Kwalombota M (2002). The effect of pregnancy in HIV-infected women. AIDS Care.

[56] Tibebu NS et al. *Depression, anxiety and stress among HIV-positive pregnant women in Ethiopia during the COVID-19 pandemic*. Trans R Soc Trop Med Hyg, 2022. 10.1093/trstmh/trac126 36579933

[57] Alio AP (2015). Paternal involvement and fetal morbidity outcomes in HIV/AIDS: a population-based study. Am J Mens Health.

[58] Hatcher AM (2021). Longitudinal association between intimate partner Violence and viral suppression during pregnancy and postpartum in South African women. Aids.

[59] Rodkjaer L (2010). Depression in patients with HIV is under-diagnosed: a cross-sectional study in Denmark. HIV Med.

[60] Mikšić Å (2018). Depression and suicidality during pregnancy. Psychiatr Danub.

[61] Caballero-Suárez NP (2017). Comparison of levels of anxiety and depression between women and men living with HIV of a Mexico City clinic. Salud Mental.

[62] Kesande C (2023). Prevalence and factors associated with psychological distress among pregnant and non-pregnant youth living with HIV in rural Uganda: a comparative study. Psychol Health Med.

[63] Wolff L, Alvarado CR M, and, Wolff M, Prevalencia R., *factores de riesgo y manejo de la depresión en pacientes con infección por VIH: Revisión de la literatura* Revista chilena de infectología, 2010. 27: p. 65–74. 20140318

[64] Moos RH, Schaefer JA. *Coping resources and processes: Current concepts and measures* 1993.

[65] Alder J (2007). Depression and anxiety during pregnancy: a risk factor for obstetric, fetal and neonatal outcome? A critical review of the literature. J Matern Fetal Neonatal Med.

[66] Hoffman S, Hatch MC (2000). Depressive symptomatology during pregnancy: evidence for an association with decreased fetal growth in pregnancies of lower social class women. Health Psychol.

[67] Ravid E (2018). Is there an association between maternal anxiety propensity and pregnancy outcomes?. BMC Pregnancy Childbirth.

[68] Lara-Cinisomo S, Griffin BA (2007). Factors associated with major depression among mothers in Los Angeles. Womens Health Issues.

[69] Dubber S (2015). Postpartum bonding: the role of perinatal depression, anxiety and maternal-fetal bonding during pregnancy. Arch Womens Ment Health.

[70] Nakic Rados S, Tadinac M, Herman R (2018). Anxiety during pregnancy and Postpartum: Course, Predictors and Comorbidity with Postpartum Depression. Acta Clin Croat.

[71] Rusanen E (2021). Prenatal expectations and other psycho-social factors as risk factors of postnatal bonding disturbance. Infant Ment Health J.

[72] Stanton AL (2000). Coping through emotional approach: scale construction and validation. J Pers Soc Psychol.

[73] Brown MJ (2020). Ways of coping and perceived HIV-related stigma among people living with HIV: moderation by sex and sexual orientation. Psychol Health Med.

